# Language Barriers and Access to Hospital Patient Portals in the US

**DOI:** 10.1001/jamanetworkopen.2025.37864

**Published:** 2025-10-16

**Authors:** Debbie W. Chen, Maya Watanabe, Steven Xie, Hattie H. Huston-Paterson, Mousumi Banerjee, Megan R. Haymart

**Affiliations:** 1 Division of Metabolism, Endocrinology & Diabetes, University of Michigan, Ann Arbor; 2 Center for Biostatistics in AIDS Research, Harvard T.H. Chan School of Public Health, Boston, Massachusetts; 3 Department of Surgery, University of Michigan, Ann Arbor; 4 Department of Surgery, David Geffen School of Medicine, University of California, Los Angeles; 5 Department of Biostatistics, University of Michigan, Ann Arbor

## Abstract

**Importance:**

Although patient portals are becoming important tools in health care delivery, there is a lack of published data on the language accessibility of these digital platforms.

**Objective:**

To examine the language accessibility of hospitals’ patient portals.

**Design, Setting, and Participants:**

This cross-sectional study identified 51 counties in the 17 US states (5 western states, 2 midwestern, 4 northeastern, and 6 southern) with at least 300 000 residents with limited English proficiency. From eligible hospitals with public-facing patient portal platforms, the following data were obtained between September and December 2024: the URL (uniform resource locator) and language accessibility of the patient portal (ie, of the login prompts), portal vendor, and whether the availability of secure portal messaging was explicitly described.

**Main Outcomes and Measures:**

The primary outcome was language accessibility of the portal login page (binary variable: only English vs in at least 2 languages). Multivariable logistic regression analysis was performed to determine factors associated with accessibility of the portal login page in at least 2 languages.

**Results:**

Among 514 eligible hospitals (194 [38.0%] in West census region), 511 (99.4%) had public-facing patient portal platforms and were thus included in the analytic cohort; 429 (84.0%) were nonteaching hospitals. Patient portals were accessible in only English at 150 hospitals (29.4%); in English and Spanish only at 305 hospitals (59.7%); and in English, Spanish, and at least 1 additional language at 56 hospitals (10.9%). Only 24 of the 511 hospitals (4.7%) offered portal access in the most common non-English, non-Spanish language of their respective counties. In multivariable logistic regression analysis, teaching hospitals had higher odds of offering portals with multilingual accessibility (odds ratio [OR], 2.21; 95% CI, 1.66-2.95) compared with nonteaching hospitals, while hospitals that used vendors other than Epic MyChart or Cerner had lower odds (OR 0.34; 95% CI, 0.24-0.48 vs Epic).

**Conclusions and Relevance:**

In this cross-sectional study of hospitals in 17 states, only a small proportion offered patient portals that were accessible in a non-English, non-Spanish language. As digital health care continues to evolve, prioritizing language accessibility in its design is essential to ensure inclusivity for all patients.

## Key Points

**Question:**

Do hospitals offer patient portals with multilingual accessibility?

**Findings:**

In this cross-sectional study of 514 hospitals across 51 counties in 17 states, 99.4% offered patient portals; 29.4% of these provided access only in English, 59.7% provided access in English and Spanish only, 10.9% provided access in 3 or more languages, and only 4.7% offered portal access in the most common non-English, non-Spanish language of their respective counties. Multivariable logistic regression found that teaching (vs nonteaching) hospitals had higher odds of offering portals with multilingual access.

**Meaning:**

These findings suggest substantial gaps persist that hinder portal accessibility for linguistically marginalized patient populations.

## Teaser

This cross-sectional study investigates the language accessibility of hospitals’ patient portals and examines whether multilingual accessibility is associated with nonclinical factors, such as hospital teaching status.

## Introduction

The 2009 Health Information Technology for Economic and Clinical Health Act incentivized health care organizations to digitize medical records and adopt the use of patient portals.^1,2^ The US government defines a patient portal as, “a secure online website that gives patients convenient 24-hour access to personal health information from anywhere with an Internet connection.”^3^ In 2017, more than 90% of health care systems offered their patients access to a patient portal.^4,5^ Although the patient portal is becoming an important tool in health care delivery, studies have consistently identified disparities in its use, with vulnerable patient populations, including patients with limited English proficiency (LEP), less likely to use the patient portal.^4,6,7,8,9^

Policies and legislation, including section 1557 of the Patient Protection and Affordable Care Act and the US Department of Health and Human Services Language Access Plan,^10,11^ are in place to support meaningful access to health care services for patients with LEP. In the US, the population of more than 25 million individuals who speak English less than very well are categorized as having LEP.^12^ Among the limited English proficient population, the most commonly spoken languages are Spanish, Chinese, and Vietnamese.^12^ However, these federal policies do not specifically address digital health care access. Additionally, granular data on the language accessibility of patient portals are lacking. Patient portals that are only accessible in English marginalize and exclude patients with non-English language preference.^4,7,13^ At a minimum, language accessibility requires the availability of standard patient portal login prompts, such as for username and password, in the patient’s preferred language.

This cross-sectional study examined the availability and language accessibility of patient portals in a cohort of hospitals across 51 counties in 17 states and identified factors associated with multilingual access. We hypothesized that many hospitals would have patient portals that are only accessible in English, and that among those with multilingual access, bilingual patient portals that are accessible in English and Spanish only would be the most common. Additionally, we posited that multilingual accessibility would be associated with nonclinical factors, such as hospital teaching status.

## Methods

### Sampling Methods

**Figure 1.**
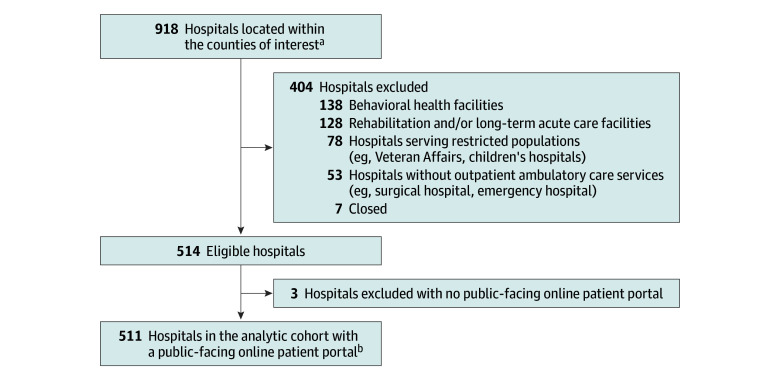
Flow Diagram of Hospital Sampling Hospitals located within the 51 counties of interest were identified from the American Hospital Association Annual Survey database. ^a^The 51 counties of interest appear in the Methods section. ^b^The number of hospitals from each state is as follows: 1 to 20 hospitals in Colorado, Georgia, North Carolina, New Jersey, New York, and Virginia; 21 to 40 hospitals in Massachusetts, Maryland, Michigan, Nevada, Pennsylvania, and Washington; and more than 40 hospitals in Arizona, California, Florida, Illinois, and Texas.

To evaluate whether the language accessibility of hospitals’ patient portal differs across regions and to oversample hospitals that are more likely to serve patients with non-English language preference, this cross-sectional study identified states that have at least 300 000 residents with LEP, based on US census data available in September 2024.^14^ This approach identified 17 US states (34%) among which the median number of residents with LEP was 522 952 (simple range, 301 467-6 799 270 residents) and the median proportion of residents with LEP was 8.9% (range, 3.2%-19.4%) (Figure 1).^14^

We then identified the 3 counties with the largest number of residents with LEP within each of the 17 states (Arizona: Maricopa, Navajo, and Pima Counties; California: Los Angeles, Orange, and San Diego Counties; Colorado: Arapahoe, Denver, and El Paso Counties; Florida: Broward, Miami-Dade, and Palm Beach Counties; Georgia: Dekalb, Fulton, and Gwinnett Counties; Illinois: Cook, DuPage, and Kane Counties; Maryland: Baltimore, Montgomery, and Prince George’s Counties; Massachusetts: Essex, Middlesex, and Suffolk Counties; Michigan: Macomb, Oakland, and Wayne Counties; Nevada: Carson City, Clark, and Washoe Counties; New Jersey: Bergen, Hudson, and Middlesex Counties; New York: Bronx, Kings, and Queens Counties; North Carolina: Guilford, Mecklenburg, and Wake Counties; Pennsylvania: Lancaster, Montgomery, and Philadelphia Counties; Texas: Dallas, Harris, and Tarrant Counties; Virginia: Fairfax, Loudoun, and Prince William Counties; and Washington: King, Pierce, and Snohomish Counties).^14^ We obtained a comprehensive list of all hospitals located within the 51 counties of interest from the American Hospital Association (AHA) Annual Survey database. Similar to cohort selection strategies implemented in prior studies,^15,16^ on the basis of information provided by the hospital’s public-facing website, we excluded rehabilitation and long-term acute-care hospitals, behavioral health facilities, hospitals serving restricted populations (ie, children’s hospitals, Veteran Affairs, and Indian Health Services), hospitals without outpatient ambulatory care services, and hospitals that had closed.

This cross-sectional study follows the Strengthening the Reporting of Observational Studies in Epidemiology (STROBE) reporting guideline. The University of Michigan Institutional Review Board deemed this study not regulated because it does not collect identifiable private information about individual members, employees, or staff of the organization that is the subject of this research.

### Variables of Interest

Between September and December 2024, we collected the following information from each of the hospitals’ public-facing patient portal platforms: the uniform resource locator (URL), the language accessibility of the patient portal login page (eg, of the login prompts), and the patient portal vendor. To better understand the role of contemporary hospital patient portal platforms in facilitating the delivery of telehealth services—particularly, secure communication between patients and their health care teams—we also collected information on whether the hospital website explicitly described the option for patients to communicate with their health care team through secure portal messaging (binary variable: yes vs no).^17,18,19,20^ Primary outcome was language accessibility of the patient portal login page (binary variable: only English vs in at least 2 languages).

Data on locality-based attributes for each of the hospitals were sourced from US federal agencies’ public-facing websites. States were categorized into US census regions (West, Midwest, Northeast, and South).^21^ To explore the potential association between the interpreter and/or translator employment landscape and language accessibility of hospital portals, we examined 2 state-level variables. Specifically, we obtained data from the US Bureau of Labor and Statistics national occupational employment and wage estimates (May 2023) on the number of interpreters and/or translators employed per 1000 jobs, and the annual mean wage for interpreters and/or translators.^22^

For the hospitals in the analytic cohort, we examined whether the language accessibility of their patient portals aligns with the linguistic needs of county residents with LEP. We obtained county-level language-based attributes data from the 2023 American Community Survey 5-year estimates on (1) the proportion of residents with LEP and of (2) the proportion of Spanish-speaking residents with LEP. We categorized both variables as small (below the national average [8.6% with LEP, and 5.6% Spanish-speaking with LEP]), moderate (8.6%-17.3% with LEP and 5.6%-11.2% Spanish-speaking with LEP), and large (greater than twice the national average [17.3% with LEP and 11.2% Spanish-speaking with LEP]).^14,23^

Previous research has shown that the availability of language-based services, such as language interpreters, in hospitals varied according to hospital characteristics like teaching status.^24^ To explore whether this association also applies to the language accessibility of hospital patient portals, hospital and/or health system members of the Association of American Medical Colleges (AAMC) were classified as teaching hospitals.^25^ Additionally, since hospital ownership status can influence hospital priorities and approaches to patient care,^26^ we obtained hospital ownership data (not-for-profit vs for-profit vs government-owned) from the AHA Annual Survey database.

### Statistical Analysis

We generated descriptive statistics for all categorical variables. χ^2^ Tests were used to test for unadjusted associations between the factors of interest and the primary outcome. Multivariable logistic regression was used to examine the association between the primary outcome (accessibility of the patient portal login page in at least 2 languages) and the following variables: employment of interpreters and/or translators, hospital teaching status, and patient portal vendor. In the model, the employment of interpreters and/or translators was defined as the number of interpreters and/or translators employed per 5000 jobs in each geographic area and was calculated as the median number of interpreters and/or translators employed (per state) across the 17 states included in the cohort, aggregated by US census region: West (Arizona, California, Colorado, Nevada, and Washington), Midwest (Illinois and Michigan), Northeast (Massachusetts, New Jersey, New York, and Pennsylvania), and South (Florida, Georgia, Maryland, North Carolina, Texas, and Virginia).^21^ Since there was collinearity between hospital ownership and hospital teaching status (*P* < .001), and prior work has focused on hospital teaching status and language access,^24^ only hospital teaching status was included in the model. We used generalized estimating equations (GEE) to account for clustering of hospitals within states. Robust (sandwich) SEs were used to obtain correct inference. To address quasicomplete data separation by state, the included states were grouped according to US census region and used as the GEE clustering variable. Factors were assessed using 2-sided hypotheses at a 5% type I error level. Statistical analyses were performed using R software version 4.2.1 (R Project for Statistical Computing).

## Results

### Language Accessibility of Hospital Patient Portals

**Figure 2.**
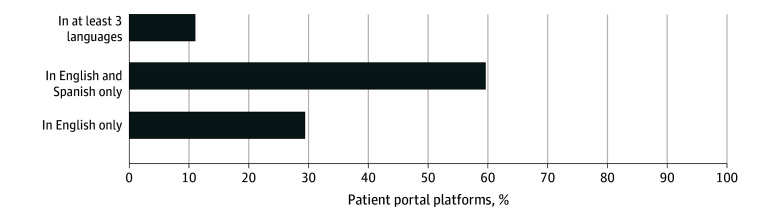
Language Accessibility of Hospitals’ Patient Portals

Of 514 eligible hospitals, 511 (99.4%) had a patient portal (the 3 hospitals that did not provide an online portal for patients to access their personal health information were excluded from the analytic cohort); 429 (84.0%) were nonteaching hospitals (Figure 1). Almost one-third of the hospitals (150 of 511 [29.4%]) had patient portals that were accessible in English only, 305 (59.7%) had portals that were accessible in English and Spanish only, and 56 (10.9%) had portals that were accessible in English, Spanish, and at least 1 other language (Arabic, Chinese, Bengali, Creole, French, Korean, Polish, Portuguese, Russian, and/or Turkish) (Figure 2). With respect to the linguistic needs of its county residents, only 24 of the 511 hospitals (4.7%) offered portal access in the most common non-English, non-Spanish language of their respective counties. In terms of functionality, most hospitals (389 of 511 [76.1%]) explicitly described on their website the option for patients to communicate with their health care team through secure portal messaging.

Within some health care systems, multiple hospitals in the same state shared a single patient portal platform that was accessible through a common URL. Overall, there were 216 distinct patient portal platforms with unique URLs. A sensitivity analysis of 216 hospitals representative of these 216 distinct portal platforms yielded similar results, with almost one-third of hospitals (63 of 216 hospitals [29.2%]) offering accessibility to its patient portal in only English, 137 (63.4%) offering accessibility in English and Spanish only, and 16 (7.4%) offering accessibility in English, Spanish, and at least 1 other language.

### Factors Associated With Hospitals Offering Portals With Multilingual Accessibility

Table 1 provides the distribution of state, county, and hospital level characteristics of the 511 hospitals with a patient portal. States with higher employment of interpreters and/or translators had a higher proportion of hospitals with portals that were accessible in at least 2 languages (high, 107 hospitals [88.4%]; intermediate, 169 hospitals [70.7%]) compared with those in states with lower employment (low, 85 hospitals [56.3%]). A higher proportion of teaching hospitals vs nonteaching hospitals (69 hospitals [84.1%] vs 292 hospitals [68.1%]) had a patient portal that was accessible in at least 2 languages. Similarly, differences were observed on the basis of hospital ownership status, with a higher proportion of government-owned (47 hospitals [88.7%]) and not-for-profit hospitals (257 hospitals [73.2%]) offering portals with multilingual accessibility than for-profit hospitals (57 hospitals [53.3%]). Additionally, a higher proportion of hospitals that used Cerner (67 hospitals [85.9%]) or Epic MyChart (203 hospitals [77.5%]) offered portals with multilingual accessibility than hospitals that used other vendors (91 hospitals [53.2%]). A higher proportion of residents with LEP in a county and a higher proportion of Spanish-speaking residents with LEP in a county were not associated with a higher proportion of hospitals offering portals with multilingual access.

**Table 1.** Univariate Analysis of Factors Associated With Accessibility of Hospital Patient Portals in at Least 2 Languages

Factors	No. of hospitals (N = 511)	Patient portals with accessibility, No. (%)		*P* value
		In only English (n = 150 [29.4%])	In ≥2 languages (n = 361 [70.6%])	
US census region				
West	194	55 (28.4)	139 (71.6)	.27
South	153	47 (30.7)	106 (69.3)	
Northeast	84	19 (22.6)	65 (77.4)	
Midwest	80	29 (36.3)	51 (63.7)	
State-level employment of interpreters and/or translators^a^				
Low	151	66 (43.7)	85 (56.3)	<.001
Intermediate	239	70 (29.3)	169 (70.7)	
High	121	14 (11.6)	107 (88.4)	
State-level annual mean wage for interpreters and/or translators, $^b^				
≤58 533	242	68 (28.1)	174 (71.9)	.62
>58 533	269	82 (30.5)	187 (69.5)	
County-level population of residents with limited English proficiency^c^				
Small	128	32 (25.0)	96 (75.0)	.08
Moderate	197	69 (35.0)	128 (65.0)	
Large	186	49 (26.3)	137 (73.7)	
Hospital teaching status				
Nonteaching	429	137 (31.9)	292 (68.1)	.005
Teaching	82	13 (15.9)	69 (84.1)	
Hospital ownership status				
Not-for-profit	351	94 (26.8)	257 (73.2)	<.001
For-profit	107	50 (46.7)	57 (53.3)	
Government-owned	53	6 (11.3)	47 (88.7)	
Patient portal vendor				
Epic MyChart	262	59 (22.5)	203 (77.5)	<.001
Other	171	80 (46.8)	91 (53.2)	
Cerner	78	11 (14.1)	67 (85.9)	

^a^ State-level employment of interpreters and/or translators was categorized as low (0.09-0.24 per 1000 jobs), intermediate (0.25-0.40 per 1000 jobs), and high (0.41-0.56 per 1000 jobs).

^b^ Annual mean wage for interpreters and/or translators was dichotomized as less than or equal to, or greater than $58 533, which was the mean wage for interpreters and/or translators across all 50 US states.

^c^ County-level population of residents with limited English proficiency was categorized as small (below the national average, 8.6%), moderate (between 8.6% and 17.3%), and large (greater than twice the national average, 17.3%).

Table 2 displays results of the multivariable logistic regression obtained using GEE. Teaching hospitals had higher odds of offering patient portals that were accessible in at least 2 languages compared with nonteaching hospitals (odds ratio, 2.21; 95% CI, 1.66-2.95). Compared with hospitals using Epic MyChart, hospitals that used other vendors had lower odds of offering portals that were accessible in at least 2 languages (odds ratio, 0.34; 95% CI, 0.24-0.48), while there was no difference in odds for hospitals that used Cerner. The employment of interpreters and/or translators, which was aggregated across geographic areas in the multivariable model, was no longer found to be associated with accessibility of hospitals’ patient portals in at least 2 languages. Sensitivity analysis of the 216 distinct patient portal platforms (eg, with unique URLs) in a similar multivariable logistic regression model yielded similar results (eTable in Supplement 1).

**Table 2.** Results of the Multivariable Logistic Regression of Factors Associated With Accessibility of Hospitals’ Patient Portals in at Least 2 Languages

Factors	OR (95% CI)
Employment of interpreters and/or translators^a^	1.47 (0.97-2.24)
Hospital teaching status	
Nonteaching	1.00 [Reference]
Teaching	2.21 (1.66-2.95)
Patient portal vendor	
Epic MyChart	1.00 [Reference]
Cerner	1.83 (0.46-7.29)
Other	0.34 (0.24-0.48)

Abbreviation: OR, odds ratio.

^a^ The OR represents the change in odds of the hospital having a patient portal that is accessible in at least 2 languages for an increase of 1 interpreter and/or translator employed per 5000 jobs.

## Discussion

This cross-sectional study highlights how gaps in the design of patient portals can disproportionately limit access to health information platforms for linguistically marginalized patient populations. Only a small proportion of the hospitals (56 hospitals [10.9%]) had patient portals that were accessible in a non-English, non-Spanish language, and almost one-third (150 hospitals [29.4%]) were accessible in only English. Furthermore, the availability of non-English language options for hospitals’ patient portals did not consistently correspond with the local demand for such services, as indicated by the county-level proportion of individuals with LEP. In our study, only 24 hospitals (4.7%) offered portal access in the 2 most common non-English languages of their respective counties.

In our study cohort, most hospitals (389 hospitals [76.1%]) explicitly described on their websites the option for patients to communicate with their health care team through secure portal messaging. This reflects a paradigm shift in health care delivery since the introduction of the first patient portal in 1999, when portal functionality was limited primarily to providing patients with electronic access to their personal health information.^27^ Since the COVID-19 pandemic in 2020, many hospitals have utilized existing technologies like the patient portal platform to provide telehealth services, which includes secure messaging and virtual video visits.^20,28^ Patient-initiated portal messages have increased exponentially since 2020, and are becoming a standard method of health care communication for some patients.^29,30^ However, some of the initial barriers that limited portal access for linguistically marginalized patient populations, including lack of broadband internet access and digital literacy challenges,^4,31,32,33^ remain unresolved. As a result, many patients continue to be excluded, even as the use of patient portals has become an increasingly integral tool in health care delivery.^34,35,36^ In today’s digital age where patient portals often serve as the front door to telehealth services, ensuring multilingual portal access is a critical first step for inclusive and equitable health care delivery.^4,37,38,39^

Since 2011, when the first bilingual English and Spanish patient portal was introduced,^40^ the US population has become more linguistically diverse. Today, more than 41 million individuals (13.3% of the US population) speak Spanish—the most common non-English language in 47 states—and more than 26 million individuals (8.4% of the US population) speak a language other than English or Spanish.^12,41,42^ Although many hospital patient portal login webpages are accessible in both English and Spanish, fewer than 1 in 20 (4.7%) are accessible in the most common non-English, non-Spanish language of their respective county. Achieving equitable health care access will require an intentional redesign of patient portals to support meaningful engagement by patients with both English and non-English language preferences, beginning with multilingual accessibility at the portal login page. This study underscores the ongoing need to expand language accessibility in patient portal access, in concert with broader efforts to improve the availability and accessibility of language-based resources in the clinical setting,^43,44^ to better serve the nation’s linguistically diverse patient population.

Alarmingly, in our study, hospitals in counties with higher proportions of residents with LEP, per US census data, were not more likely to have a patient portal that was accessible in at least 2 languages, despite the need. These findings likely reflect the intersection of multiple factors, including state-level priorities surrounding language access, the availability of language-based resources, and the perceived buying power of racially and ethnically minoritized groups.^45^

Eighty percent of hospitals in the US are not teaching hospitals.^46^ Consistent with this representation, our cohort included 429 (84.0%) nonteaching and 82 (16.0%) teaching hospitals. Our findings are complementary to a prior study from Prada-Rey et al^47^ that focused exclusively on the patient portal login pages of AAMC-member teaching hospitals, which represent only 5% of all US hospitals.^48^ Prada-Rey et al^47^ found that 33.7% of the teaching hospitals had patient portals that were only accessible in English, and 23.2% had portals that were accessible in a language other than English or Spanish. Our study builds upon this prior work with a more heterogeneous hospital cohort that includes nonteaching hospitals, which constitute the majority of hospital types in the US,^49^ and municipal hospitals.^50^

Just as hospitals have successfully leveraged existing health information technologies to provide telehealth services, they can utilize existing digital health infrastructures to start to improve the language accessibility of their patient portal platforms. Some, but not all, of the hospitals that used the 2 major patient portal vendors in this study, which offer translation of standard content such as website headers and navigation links, had multilingual accessibility to their patient portal platforms.^51,52^ There is an opportunity for other hospitals and health systems that use these vendors to do the same and expand access of their patient portals to a more linguistically diverse patient population.

### Strengths and Limitations

Strengths of our study include the inclusion of a large heterogeneous cohort of both teaching and nonteaching hospitals located across 17 states with varied proportions of residents with LEP. Second, our study focused on language accessibility of the patient portal login page, which serves as the digital front door to accessing any and all of the functionalities of the patient portal, including telehealth services. Additionally, the sensitivity analysis of 216 hospitals with distinct patient portal platforms (eg, with unique URLs) yielded results similar to those obtained from the analysis of the full cohort of 511 hospitals.

Some potential limitations should be noted. First, the focus of this study was on evaluating language accessibility of the patient portal login page (ie, prompts for username and password). Although assessing login capabilities is an important first step, additional next steps include exploring the language-based usability of these platforms beyond the login page. Second, the study cohort was restricted to hospitals in the states with at least 300 000 residents with LEP, according to US census data.^14^ Therefore, our study findings may not be generalizable to hospital in states with smaller population of residents with LEP, which may be less likely to offer a patient portal with multilingual accessibility. Third, there was a statistical need to aggregate employment data for interpreters and/or translators across geographic areas in the multivariable logistic regression analysis, which may have contributed to less specificity and, therefore, a lack of a statistically significant association in the multivariable model compared with the unadjusted analysis. Fourth, we only assessed whether secure messaging is an explicitly described feature of the patient portal, and as such, we did not evaluate the availability of language-based support for, or the patient experience in, sending a secure portal message.

## Conclusions

In this cross-sectional study of hospitals across 51 counties in one-third of US states, we highlighted a significant gap in the accessibility of hospitals’ patient portals due to language, which limits patients with non-English language preference from fully participating in their health care. As patient portals become increasingly integral to health care delivery, targeted interventions are necessary to build more inclusive health care platforms that promote greater access and equity for all patients. Without these improvements, linguistically marginalized patient populations will remain at a disadvantage in accessing and utilizing health care information and services.

## Notes

Supplement 1. **eTable.** Results of the multivariable logistic regression of factors associated with accessibility of hospitals’ patient portals in at least 2 languages in the sensitivity analysis of the 216 distinct patient portal platforms

Supplement 2.
Data Sharing Statement

## References

[1] Kruse CS, Bolton K, Freriks G. The effect of patient portals on quality outcomes and its implications to meaningful use: a systematic review. J Med Internet Res. 2015;17(2):e44. doi:10.2196/jmir.3171 25669240 PMC4342639

[2] Neuner J, Fedders M, Caravella M, Bradford L, Schapira M. Meaningful use and the patient portal: patient enrollment, use, and satisfaction with patient portals at a later-adopting center. Am J Med Qual. 2015;30(2):105-113. doi:10.1177/1062860614523488 24563085 PMC4141030

[3] Office of the National Coordinator for Health Information Technology (ONC). What is a patient portal? Accessed March 26, 2025. https://www.healthit.gov/faq/what-patient-portal#:~:text=A%20patient%20portal%20is%20a,Discharge%20summaries

[4] Rodriguez JA, Casillas A, Cook BL, Marlin RP. The language of equity in digital health: prioritizing the needs of limited English proficient communities in the patient portal 2.0. J Health Care Poor Underserved. 2021;32(2):211-219. doi:10.1353/hpu.2021.0059

[5] Heath S. Patient portal adoption tops 90%, but strong patient use is needed. Tech Target. 2018. Accessed March 26, 2025. https://www.techtarget.com/patientengagement/news/366585192/Patient-Portal-Adoption-Tops-90-But-Strong-Patient-Use-Is-Needed

[6] Khatib R, Glowacki N, Chang E, Lauffenburger J, Pletcher MJ, Siddiqi A. Disparities in patient portal engagement among patients with hypertension treated in primary care. JAMA Netw Open. 2024;7(5):e2411649. doi:10.1001/jamanetworkopen.2024.11649 38748420 PMC11096988

[7] Casillas A, Abhat A, Vassar SD, . Not speaking the same language-lower portal use for limited English proficient patients in the Los Angeles safety net. J Health Care Poor Underserved. 2021;32(4):2055-2070. doi:10.1353/hpu.2021.0182 34803059

[8] Gordon NP, Torreblanca A, Ford RG, Ou S, Lin MW. Lower use of and potential barriers to using patient portals among limited English proficient Latino and Chinese American adults: a health techquity concern. Perm J. 2025;29(1):1-22. doi:10.7812/TPP/24.119 39935330 PMC11907665

[9] Elston Lafata J, Miller CA, Shires DA, Dyer K, Ratliff SM, Schreiber M. Patients’ adoption of and feature access within electronic patient portals. Am J Manag Care. 2018;24(11):e352-e357. 30452203 PMC6613379

[10] US Department of Health and Human Services. Language access plan, fiscal year 2024. Accessed March 26, 2025. https://aspe.hhs.gov/sites/default/files/documents/db5a4bb6b13d359b31baf50bdde9c8cb/aspe-language-access-plan.pdf

[11] US Department of Health and Human Services. Section 1557: frequently asked questions. Accessed August 1, 2025. https://www.hhs.gov/sites/default/files/section-1557-final-rule-faqs-7282017rev15.pdf

[12] US Census Bureau. Detailed languages spoken at home and ability to speak English for the population 5 years and over: 2017-2021. 2025. Accessed August 1, 2025.https://www.census.gov/data/tables/time-series/demo/language-use/2017-2021-lang-tables.html

[13] Localio AM, Klusaritz H, Morales KH, Ruggieri DG, Han X, Apter AJ. Primary language and the electronic health record patient portal: barriers to use among Spanish-speaking adults with asthma. J Asthma. 2022;59(10):2081-2090. doi:10.1080/02770903.2021.1989462 34634975

[14] US Census Bureau. Detailed languages spoken at home and ability to speak English for the population: 5 years and over: 2009-2013. 2015. Accessed September 1, 2024. https://www.census.gov/data/tables/2013/demo/2009-2013-lang-tables.html

[15] Chen DW, Banerjee M, Gay B, . Access to new clinic appointments for patients with cancer. JAMA Netw Open. 2024;7(6):e2415587. doi:10.1001/jamanetworkopen.2024.15587 38848062 PMC11161839

[16] Chen DW, Banerjee M, He X, . Hidden disparities: how language influences patients’ access to cancer care. J Natl Compr Canc Netw. 2023;21(9):951-959.e1. doi:10.6004/jnccn.2023.7037 37673110 PMC11033703

[17] US Government Accountability Office. Electronic health information exchange: use has increased, but is lower for small and rural providers. 2023. Accessed September 5, 2025. https://www.gao.gov/assets/gao-23-105540.pdf

[18] Gordon NP, Yin C, Lo JC. Examining whether patient portal and video visit use differs by race and ethnicity among older adults in a us integrated health care delivery system: cross-sectional electronic health record and survey-based study. JMIR Aging. 2024;7:e63814. doi:10.2196/63814 39509698 PMC11582487

[19] Tang M, Mishuris RG, Payvandi L, Stern AD. Differences in care team response to patient portal messages by patient race and ethnicity. JAMA Netw Open. 2024;7(3):e242618. doi:10.1001/jamanetworkopen.2024.2618 38497963 PMC10949096

[20] Telehealth.HHS.gov. What are different types of telehealth? 2024. Accessed September 3, 2025. https://telehealth.hhs.gov/patients/what-are-different-types-telehealth

[21] US Census Bureau. Census regions and divisions of the United States. Accessed September 3, 2025. https://www2.census.gov/geo/pdfs/maps-data/maps/reference/us_regdiv.pdf

[22] US Bureau of Labor Statistics. Occupational employment and wage statistics, May 2023. 2024. Accessed September 2, 2025. https://www.bls.gov/oes/current/oes273091.htm#st

[23] US Census Bureau. C16001: language spoken at home for the population 5 years and over. Accessed January 1, 2025. https://data.census.gov/table/ACSDT5Y2023.C16001

[24] Hasnain-Wynia R, Yonek J, Pierce D, Kang R, Greising CH. Hospital language services for patients with limited English proficiency: results from a national survey. National Health Law Program. 2006. Accessed September 3, 2025. https://healthlaw.org/wp-content/uploads/2018/09/HRET.Language.Services.pdf

[25] Association of American Medical Colleges. Member directory. Accessed March 26, 2025. https://myengagement.aamc.org/memberdirectory

[26] Horwitz JR. Making profits and providing care: comparing nonprofit, for-profit, and government hospitals. Health Aff (Millwood). 2005;24(3):790-801. doi:10.1377/hlthaff.24.3.790 15886174

[27] Halamka JD, Mandl KD, Tang PC. Early experiences with personal health records. J Am Med Inform Assoc. 2008;15(1):1-7. doi:10.1197/jamia.M2562 17947615 PMC2274878

[28] Patel PD, Cobb J, Wright D, . Rapid development of telehealth capabilities within pediatric patient portal infrastructure for COVID-19 care: barriers, solutions, results. J Am Med Inform Assoc. 2020;27(7):1116-1120. doi:10.1093/jamia/ocaa065 32302395 PMC7188108

[29] Gold KJ, Chen D, Shumer G, . Patient-reported reasons for sending portal messages: a survey of use in a family medicine department. J Gen Intern Med. 2024;39(13):2608-2611. doi:10.1007/s11606-024-08815-6 38831243 PMC11436532

[30] Holmgren AJ, Sinsky CA, Rotenstein L, Apathy NC. National comparison of ambulatory physician electronic health record use across specialties. J Gen Intern Med. 2024;39(14):2868-2870. doi:10.1007/s11606-024-08930-4 38980460 PMC11534958

[31] Chen DW, Reyes-Gastelum D, Hawley ST, Wallner LP, Hamilton AS, Haymart MR. Unmet information needs among Hispanic women with thyroid cancer. J Clin Endocrinol Metab. 2021;106(7):e2680-e2687. doi:10.1210/clinem/dgab128 33660770 PMC8208677

[32] Pew Research Center. Internet use among Hispanics. 2016. Accessed July 28, 2025. https://www.pewresearch.org/race-and-ethnicity/2016/07/20/1-internet-use-among-hispanics/

[33] Lee G, Chang A, Pal A, Tran TA, Cui X, Quach T. Understanding and addressing the digital health literacy needs of low-income limited English proficient Asian American patients. Health Equity. 2022;6(1):494-499. doi:10.1089/heq.2022.0045 36186613 PMC9518790

[34] Guetterman TC, Koptyra E, Ritchie O, . Equity in virtual care: a mixed methods study of persepctives from physicians. J Telemed Telecare. 2025;31(3):408-416.doi:10.1177/1357633X231194382 37641207

[35] Dia M, Davoudi S, Sanayei N, . Demographic and socioeconomic disparities in the hybrid ophthalmology telemedicine model. J Telemed Telecare. 2025;31(5):697-704. doi:10.1177/1357633X231211353 37960873

[36] Bharadwaj M, Langbein B, Labban M, Lipsitz SR, Licurse AM, Trinh QD. Patterns and disparities in telehealth usage during the COVID-19 pandemic across surgical specialties. Telemed J E Health. 2024;30(3):866-873. doi:10.1089/tmj.2022.0332 37699226

[37] Rodriguez JA, Shachar C, Bates DW. Digital inclusion as health care—supporting health care equity with digital-infrastructure initiatives. N Engl J Med. 2022;386(12):1101-1103. doi:10.1056/NEJMp2115646 35302722

[38] Craig S, Shen A, Wallis K, . How health systems can help address language barriers to achieve digital health equity. 2021. Accessed March 28, 2025. https://ldi.upenn.edu/our-work/research-updates/how-health-systems-can-help-address-language-barriers-to-achieve-digital-health-equity/

[39] Tan-McGrory A, Schwamm LH, Kirwan C, Betancourt JR, Barreto EA. Addressing virtual care disparities for patients with limited English proficiency. Am J Manag Care. 2022;28(1):36-40. doi:10.37765/ajmc.2022.88814 35049259

[40] Office of the National Coordinatory for Health IT. Meeting the needs of a diverse patient population through patient portals. Accessed September 3, 2025. https://www.healthit.gov/case-study/meeting-needs-diverse-patient-population-through-patient-portals

[41] World Economic Forum. Besides English and Spanish, which language do you think is the most commonly spoken in the U.S.? 2021. Accessed April 2, 2025. https://www.weforum.org/stories/2021/12/spoken-language-united-states-america-english-spanish-mandarin/

[42] Centers for Medicare and Medicaid Services. Appendix a: top 15 non-English languages by state. Accessed March 26, 2025. https://www.cms.gov/cciio/resources/regulations-and-guidance/downloads/appendix-a-top-15.pdf

[43] Betancourt JR, Renfrew MR, Green AR, . Improving patient safety systems for patients with limited English proficiency: a guide for hospitals. Agency for Healthcare Research and Quality. 2012. Accessed September 8, 2025. https://www.ahrq.gov/sites/default/files/publications/files/lepguide.pdf

[44] Ortega P, Shin TM. Language is not a barrier—it is an opportunity to improve health equity through education. Health Affairs. 2021. Accessed September 3, 2025. https://www.healthaffairs.org/content/forefront/language-not-barrier-opportunity-improve-health-equity-through-education

[45] Cabrera T. The translation and interpreting industry in the United States. Informes del Observatorio / Observatorio Reports. 2017. Accessed March 27, 2025. https://cervantesobservatorio.fas.harvard.edu/sites/default/files/028_report_translation_2.pdf

[46] American Hospital Association. Teaching Hospitals vital for tomorrow’s health care. 2013. Accessed April 3, 2025. https://www.aha.org/system/files/2018-02/info-teaching.pdf

[47] Prada-Rey N, Samal L, Rodriguez JA. Language availability of patient portals at academic medical centers in the United States. J Gen Intern Med. 2025;40(6):1477-1479. doi:10.1007/s11606-024-09180-0 39500842 PMC12045902

[48] Association of American Medical Colleges. Teaching hospital characteristics. 2024. Accessed April 3, 2025. https://www.aamc.org/media/10266/download#:~:text=beds-,Overview%20of%20AAMC%2DMember%20Hospital%20Services,operate

[49] American Hospital Association. Fast facts on US hospitals. 2025. Accessed April 2, 2025. https://www.aha.org/system/files/media/file/2025/01/Fast-Facts-on-US-Hospitals-2025-Infographics.pdf

[50] Feldman AM. The municipal safety net hospital: a concept whose time has come again? Clin Transl Sci. 2011;4(1):1-2. doi:10.1111/j.1752-8062.2011.00267.x 21348946 PMC5439847

[51] EpicShare. Q: How can we reduce barriers to care for Spanish-speaking patients? 2025. Accessed March 26, 2025. https://www.epicshare.org/tips-and-tricks/spanish-mychart

[52] Markert C. A multilingual approach to EHR and patient portal translation. 2023. Accessed March 28, 2025. https://www.languageline.com/blog/a-multilingual-approach-to-ehr-and-patient-portal-translation

