# Supplementary material for: Language Barriers and Access to Hospital Patient Portals in the US
Source: JAMA Netw Open. 2025 Oct 16;8(10):e2537864. doi: 10.1001/jamanetworkopen.2025.37864 (PMC12531879; doi:10.1001/jamanetworkopen.2025.37864)
Supplement: Supplement 1. — eTable. Results of the multivariable logistic regression of factors associated with accessibility of hospitals’ patient portals in at least 2 languages in the sensitivity analysis of the 216 distinct patient portal platforms [file jamanetwopen-e2537864-s001.pdf]

Supplemental Online Content

Chen DW, Watanabe M, Xie S, Huston-Paterson HH, Banerjee M, Haymart MR. Language barriers and access to hospital patient portals in the US. *JAMA Netw Open.* 2025;8(10):e2537864. doi:10.1001/jamanetworkopen.2025.37864

eTable. Results of the multivariable logistic regression of factors associated with accessibility of hospitals' patient portals in at least 2 languages in the sensitivity analysis of the 216 distinct patient portal platforms

This supplemental material has been provided by the authors to give readers additional information about their work.

© 2025 Chen DW et al. *JAMA Network Open*.

eTable 1. Results of the multivariable logistic regression of factors associated with accessibility of hospitals' patient portals in at least 2 languages in the sensitivity analysis of the 216 distinct patient portal platforms

Sensitivity analysis of the 216 distinct patient portal platforms (i.e., with unique URLs). Results of the multivariable logistic regression of factors associated with accessibility of hospitals' patient portals in at least two languages.

	OR (95% CI)
**Employment of interpreters/translators per 5,000 jobs** (median among states within the same geographic area) [a]	
	3.50 (0.99 – 12.38)
**Patient portal vendor**	
Epic MyChart	Reference
Cerner	6.03 (0.39 – 94.34)
Other	0.38 (0.22 – 0.66)

Abbreviations: OR; odds ratio; CI, confidence interval.

[a] In this statistical model, the employment of interpreters/translators was defined as the number of interpreters/translators employed per 5,000 jobs in each geographic area and calculated as the median number of interpreters/translators employed (per state) across the 17 states included in the cohort, aggregated by US census region: West (Arizona, California, Colorado, Nevada, Washington), Midwest (Illinois, Michigan), Northeast (Massachusetts, New Jersey, New York, Pennsylvania), and South (Florida, Georgia, Maryland, North Carolina, Texas, and Virginia). The employment of interpreters/translators was assessed as a continuous variable per 5,000 jobs for ease of interpretation. Thus, the OR represents the change in odds of the hospital having a patient portal that is accessible in at least two languages for an increase of one interpreter/translator employed per 5,000 jobs.

© 2025 Chen DW et al. *JAMA Network Open*.
