# Supplementary material for: Language Barriers and Access to Hospital Patient Portals in the US
Source: JAMA Netw Open. 2025 Oct 16;8(10):e2537864. doi: 10.1001/jamanetworkopen.2025.37864 (PMC12531879; doi:10.1001/jamanetworkopen.2025.37864)
Supplement: Supplement 2. — Data Sharing Statement [file jamanetwopen-e2537864-s002.pdf]

Data Sharing Statement

Chen. Language Barriers and Access to Hospital Patient Portals in the US. *JAMA Netw Open*. Published October 16, 2025. doi:10.1001/jamanetworkopen.2025.37864

Data
Data available: Yes
Data types: Deidentified participant data, Data (not involving human participants)
How to access data: The deidentified data supporting this study will be available upon request after publication of this manuscript for research and ending 5 years following article publication.
When available: With publication

Supporting Documents
Document types: None

Additional Information
Who can access the data: Requests should be directed to corresponding author (Debbie Chen, MD at chendeb@med.umich.edu).
Types of analyses: The deidentified data supporting this study will be available upon request for research purpose.
Mechanisms of data availability: To gain access, data requestors will need to sign a data access agreement.
